# Using network analysis to model associations between psychological symptoms, sexual function, and sexual distress in women^☆^

**DOI:** 10.1016/j.ijchp.2024.100479

**Published:** 2024-06-29

**Authors:** Marta T. Kolbuszewska, Inês M. Tavares, Pedro J. Nobre, Samantha J. Dawson

**Affiliations:** a Department of Psychology, University of British Columbia, 2136 West Mall, Vancouver, BC V6T 1Z4, Canada; b Department of Psychology and Neuroscience, Dalhousie University, Halifax, Nova Scotia, Canada; c Faculty of Psychology and Educational Sciences, University of Porto, Porto, Portugal

**Keywords:** Sexual function, Sexual distress, Anxiety, Depression, Somatization

## Abstract

**Background:**

Psychological difficulties, including depression, anxiety, and somatization, are among the most important predictors for women's sexual function (i.e., arousal, desire, lubrication, pain, and satisfaction) and sexual distress. These associations have largely been studied at the construct level, with little research examining which specific symptoms might be most important for maintaining links between psychological difficulties and domains of sexual function. The present research sought to establish and characterize networks of women's psychological symptoms, sexual function, and sexual distress, and identify potential bridge symptoms that connect them.

**Methods:**

In a cross–sectional study, 725 women reported on their sexual function, sexual distress, and depressive, anxiety, and somatization symptoms. A series of network analyses was used to identify central symptoms and connections between psychological symptoms, sexual function domains, and sexual distress.

**Results:**

Across the modeled networks, sexual distress and pain during sex were consistent bridges between other sexual function domains and psychological symptoms.

**Discussion:**

Overall, our models revealed sexual distress as an important potential mediator between sexual function problems and psychological symptoms that might contribute to the development and maintenance of comorbid sexual function and psychological problems.

## Introduction

Psychological difficulties (e.g., depression, anxiety, and somatization), are some of the most important risk factors for women's sexual dysfunction (Gonçalves et al., 2023; Moyneur et al., 2020; Polland et al., 2019). Associations between depressive, anxious, and somatic symptoms and problems with domains of sexual function are particularly notable in women (e.g., Gonçalves et al., 2023; Herder et al., 2023; Liu et al., 2023). Women exhibit higher rates of sexual impairment across domains, as well as higher rates of comorbidity between psychological symptomatology and sexual function problems than men (for meta-analyses and reviews, see Briken et al., 2020; Gonçalves et al., 2023). Psychological difficulties both precede and follow distressing problems with sexual function (e.g., Gonçalves et al., 2023; Moyneur et al., 2020; Polland et al., 2019). This comorbidity has mostly been examined at the construct level with little research considering the *specific symptoms* that are most important for maintaining links between psychological difficulties and domains of sexual function. Moreover, no research to date has evaluated the precise pathways that connect psychological difficulties with sexual function and sexual distress. Examining these associations might elucidate key factors contributing to the high comorbidity between women's sexual function problems and psychological difficulties. In the current study, we use network analysis to examine which specific symptoms might be most relevant for bridging sexual function problems, sexual distress, and psychological difficulties.

### Associations between psychological symptoms and sexual function

Extensive evidence links depression and depressive symptoms with sexual function problems in all domains, including pain during sex (e.g., Pâquet et al., 2018), arousal and desire (e.g., Rosen et al., 2019), orgasm (e.g., Leeners et al., 2013), and low sexual satisfaction (e.g., Karakose et al., 2023). Indeed, between 60 % and 90 % of women with a diagnosis of depression report some problems with their sexual function (Sreelakshmy et al., 2017) and meta–analytic evidence supports bidirectional links between depression and sexual function problems (Atlantis & Sullivan, 2012).

Similarly, anxiety disorders and symptoms have been consistently linked to problems with sexual function. Most notably, anxiety is linked with pain during sex (e.g., Chisari et al., 2021; Govind et al., 2020; Tribó et al., 2020), though we see similar associations for problems with arousal and desire (e.g., Hendrickx et al., 2016, cf., Soler et al., 2021), orgasm (e.g., Hevesi et al., 2020), and low sexual satisfaction (e.g., Carcedo et al., 2020). However, longitudinal evidence for the directionality of relationships between anxiety and sexual function is scarce and findings are inconsistent (Forbes et al., 2016a; Kalmbach et al., 2015). For example, experimental evidence supports that the induction of anxiety can both increase, decrease, and have no effect on genital and self-reported sexual arousal (Kane et al., 2019; Dewitte & Meulders, 2023). These inconsistencies may in part be due to how anxiety is induced (e.g., physiological versus cognitive). Thus, there is preliminary evidence to support the importance of examining symptom level associations between anxiety and sexual function given that the various anxiety symptoms may show different associations with the various facets of sexual function.

Links between somatization problems and sexual function have received less research attention. Somatization is associated with greater pain during sex (e.g., Ferraz et al., 2023), impaired genital but not self–reported arousal (e.g., Scavello et al., 2021), lower sexual desire (e.g., Soler et al., 2021), and orgasm difficulties (e.g., Leeners et al., 2013). Compared to individuals without somatic disorders, those with somatic disorders also report significantly less sexual satisfaction (e.g., Vanwesenbeeck et al., 2014).

Sexual function problems are frequently accompanied by sexual distress. Although sexual distress is essential to diagnoses of sexual dysfunction (American Psychiatric Association, 2022), problems with sexual function can occur without associated sexual distress (Huberts et al., 2023). Importantly, sexual distress is predicted by non–sexual psychological variables (e.g., depression, anxiety, and somatization symptoms) above and beyond poor sexual function, suggesting sexual distress may be one possible path for comorbidity between sexual function problems and psychological problems (Graham et al., 2020; Hendrickx et al., 2016).

### Network analysis in psychopathology

In contrast to traditional categorical approaches that characterize psychological disorders as distinct entities that are either present or absent, recent approaches consider psychological problems from a transdiagnostic, dimensional perspective (Dalgleish et al., 2020). Prominent models of sexual dysfunction similarly emphasize transdiagnostic and psychological factors in the development and maintenance of sexual function difficulties (Pascoal et al., 2020; Rosen & Bergeron, 2019). In addition to transdiagnostic models of psychopathology that situate the importance of bridging symptoms for explaining comorbidity, how symptoms interact with one another is relevant. While traditional correlational methods can tell us the former, the latter requires a novel approach to conceptualizing relationships between symptoms: Network Analysis.

Network Analysis refers to a broad range of modelling approaches for depicting relations between objects (Epskamp & Isvoranu, 2022). Networks are structures containing nodes (i.e., vertices) and edges (i.e., the relationships between them). One way network approaches benefit the study of psychopathology is by conceptualizing psychological disorders as being upheld through symptom interactions, rather than emerging from latent unobserved constructs (McNally, 2016). Another way network analysis benefits the study of psychopathology is by providing an avenue to explore comorbidity. By modelling how symptoms of different disorders relate to one another, network analysis can explore which symptoms might bridge disorders with one another. Past network studies examining comorbidity between generalized anxiety disorder and major depressive disorder have found that symptoms like irritability and nervousness serve as transdiagnostic bridges between disorders (Beard et al., 2016; Park & Kim, 2020). Because sexual function problems (e.g., genital pain) are highly comorbid with depressive, anxiety, and somatic symptoms, identifying which symptoms are most central is highly relevant for identifying potential points of intervention. It is possible that certain sexual function and psychological symptoms may be linked more strongly than others. As such, examining these associations at the symptom rather than construct level may further elucidate the pathways linking psychological disorders to sexual function problems.

In sum, poorer function across all sexual function domains is associated with depression, anxiety, and somatization, but debate remains over which specific psychological symptoms are most relevant for sexual function problems (Witting et al., 2008). Given that some domains of sexual function and sexual distress are more strongly linked than others, identifying the importance of certain domains over others in upholding problems with sexual function is valuable for better conceptualizations of sexual dysfunction. Moreover, past research has largely used bivariate correlations to measure associations between psychological and sexual disorders; by measuring symptom-level partial correlations, the network approach enables an examination of the relationship between any two given symptoms while holding others constant, which may reveal important pathways for symptom contagion (Borsboom & Cramer, 2013; Robinaugh et al., 2016). Identification of these links may also be relevant for guiding future treatment development by targeting the symptoms most relevant to sexual dysfunction, in line with recent research testing network theory in psychopathology (Borsboom & Cramer, 2013).

### The current study

The present research establishes networks of associations between psychological symptoms, sexual function symptoms, and sexual distress in women using network analysis with the goal of further understanding the interplay of these symptoms. This study examines central symptoms that may maintain distressing problems with sexual function and models the pathways (i.e., edges) that might connect sexual function with sexual distress, as well as psychological symptoms. The study was preregistered [https://osf.io/p3vmt].

## Materials and methods

### Participants

A cross–sectional sample of Portuguese-speaking adults (*N* = 1492 participants; *N* = 1045 women) was gathered between October 2018 and January 2021. Participants had to be at least 18 and able to read Portuguese (see Table 1 for demographics). Participants who indicated no sexual activity on any item of the Female Sexual Function Index (FSFI; Rosen et al., 2000) were excluded (*n* = 246), in line with recent recommendations for scoring the FSFI (Meston et al., 2020). Additionally, women who did not complete the Brief Symptoms Inventory (BSI–18; DeRogatis, 2001) were excluded (*n* = 74). All remaining participants correctly answered two or more out of three attention checks. The final sample consisted of 725 adult women.

**Table 1:** Participant demographics.

Characteristics	*N* (%); Mean (*SD*); [range]
Age*	28.44 *(9.10)* [18–66]
**Education**	
<12 years	4 (<1 %)
12 years	183 (25 %)
Bachelor's Degree	302 (42 %)
Graduate Degree	236 (33 %)
**Employment Status**^**†**^	
Employed	333 (46 %)
Unemployed	43 (6 %)
Retired	2 (<1 %)
Student	299 (41 %)
**Religious**	
No	298 (41 %)
Yes	427 (59 %)
**Relationship Status**	
Single	142 (20 %)
Dating	386 (53 %)
Married or Common Law	175 (24 %)
Separated or Divorced	22 (3 %)
**Nationality**	
Portuguese	718 (99 %)
Brazilian	4 (<1 %)
Angolan	1 (<1 %)
Basque	1 (<1 %)
Canadian	1 (<1 %)
**Parity**	
0	586 (81 %)
1+	139 (19 %)
**Self-Reported Medical Diagnoses**	
Urogynecological Condition^††^	178 (25 %)
Cardiovascular Condition^†††^	76 (10 %)
Neurological Disorder	20 (3 %)
Chronic Pain	43 (6 %)
Diabetes	7 (1 %)
Substance Use Disorder	9 (1 %)
Hematological Condition	25 (3 %)
Cancer	6 (<1 %)
Anxiety Disorder	315 (43 %)
Depressive Disorder	153 (21 %)
**Medication Use**	
Antihypertensive	14 (2 %)
Antidepressant	87 (12 %)
Antipsychotic	6 (<1 %)
Mood Stabilizer	7 (1 %)
Hormonal Therapy/Hormonal Contraceptive	372 (51 %)
**FSDS Distress**	11.16 *(9.29)* [0–43]
**FSFI Arousal**	4.72 *(0.96)* [1.2–6.0]
**FSFI Desire**	4.34 *(1.11)* [1.2–6.0]
**FSFI Lubrication**	5.16 *(0.98)* [1.2–6.0]
**FSFI Orgasm**	4.98 *(1.13)* [1.2–6.0]
**FSFI Pain**	5.06 *(1.08)* [1.2–6.0]
**FSFI Satisfaction**	4.56 *(1.40)* [1.2–6.0]
**BSI–18 Depression**	5.16 *(4.85)* [0–23]
**BSI–18 Anxiety**	5.62 *(4.35)* [0–24]
**BSI–18 Somatization**	2.95 *(3.33)* [0–19]

*Note:* Percentages are rounded to the nearest whole number. *Mean age is based on 724 participants with valid data. ^†^Data on employment status were missing for 48 participants. ^††^Urogynecological Disorder consists of self-reported endometriosis, gynecological problem, urological problem, and/or STI. ^†††^Cardiovascular Disorder consists of self-reported hypertension, stroke, and/or heart problems.

### Procedure

Participants were recruited through social media (e.g., academic pages, sexual health newsletters), print posters (e.g., at sexual health clinics), and by referral from sexual health professionals throughout Portugal. Participants were provided an electronic link to complete an eligibility screener followed by the online survey on an anonymous platform (i.e., no IP addresses were registered). No monetary compensation was provided. All procedures were approved by the research ethics board at the University of Porto .

### Measures

#### Sexual function

Participants completed the Portuguese validated 19–item version of the FSFI (Pechorro et al., 2009; Rosen et al., 2000). The FSFI has six domain subscales assessing desire, arousal, lubrication, orgasm, satisfaction, and pain. Possible scores range from 1.2 to 6. Higher scores indicate better sexual function; as such, higher scores on the pain subscale should be interpreted as higher pain function (i.e., lower pain). The FSFI (ω_FSFI_ = 0.95) and its subscales (ω_desire_ = 0.91; ω_arousal_ = 0.86; ω_lubrication_ = 0.94; ω_orgasm_ = 0.91; ω_satisfaction_ = 0.88; ω_pain_ = 0.88) showed good reliability.

#### Sexual distress

The Portuguese validated Female Sexual Distress Scale – Revised (FSDS–R; DeRogatis et al., 2002; Tavares et al., 2022) is a 13–item measure of sexual distress. Higher scores indicate higher levels of distress. The FSDS–R demonstrated a unidimensional factor structure (internal consistency was high; ω = 0.95). Consequently, the mean score was used to capture distress in the networks, deviating from our preregistered plan to include the 13 items individually in the models.

#### Psychological symptoms

The Portuguese validated Brief Symptoms Inventory–18 (Canavarro et al., 2017; DeRogatis, 2001) is an 18–item measure of psychological distress. Higher scores indicate higher levels of psychological distress. The BSI-18 has demonstrated a 3-factor solution. In order to examine unique connections each construct has with sexual function and sexual distress, as well as to maximize power, separate analyses were conducted with each subscale. Internal consistency for the three subscales was high (ω_depression_ = 0.92; ω_anxiety_ = 0.91; ω_somatization_ = 0.82).

### Analyses and research design

Three cross–sectional networks were fit to the data capturing associations between sexual function, sexual distress, and depression, anxiety, and somatization symptoms, respectively. Networks were estimated in R using qgraph (Epskamp et al., 2012). De–identified data, code, and details regarding analyses are available online [https://osf.io/dzysc/].

## Results

**Fig. 1:**
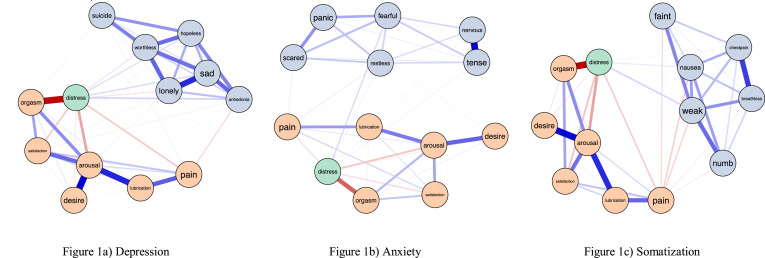
Networks of psychological symptoms, sexual function, and sexual distress *Note.* Thickness of lines denotes the strength of associations while colour denotes valence. Blue lines denote positive associations and red lines denote negative associations.

Fig. 1a–c shows connections between sexual function domains, sexual distress, and depression, anxiety, and somatization symptoms respectively. Networks were graphed using the Fruchterman–Reingold layout. Although networks cannot be interpreted solely through visual means, the visualized networks indicate valence and strength of relationships between nodes.

## Depression, sexual function, and sexual distress symptoms

### Centrality, nodes, and edges

Strength centrality and expected influence were used to assess the importance of each node to the network. Strength centrality represents how closely linked a node is to other symptoms in the network, with higher values (i.e., absolute sum of edge weights) indicating greater strength. In the depression network, arousal, sadness, worthlessness, and distress had the greatest centrality, meaning these symptoms were most strongly directly connected to other symptoms. Stability analyses revealed our network structure to be robust: The centrality stability coefficient was 0.85, indicating that 85 % of the sample size could be dropped to maintain a correlation of 0.7 (with a 95 % CI) with the original network structure.

Like strength centrality, one–step expected influence measures the direct link of a node to its neighbours (Robinaugh et al., 2016); however, expected influence accounts for both positive and negative edge weights, accounting for differential relationships symptoms may have with each other (e.g., higher sexual arousal function is positively associated with lubrication and orgasm function, but negatively associated with sexual distress and loneliness). In order, symptoms with greatest one–step expected influence were arousal, sadness, and worthlessness. Two-step expected influence takes in account not only direct connections to adjacent nodes, but also secondary indirect connections to nodes two steps away (e.g., if desire is directly connected only to arousal, but arousal is directly connected to numerous other nodes, two-step expected influence would consider these indirect influences). Sadness, worthlessness, and arousal had the greatest two-step expected influence.

A bootstrapped edge–weight difference test (*α* = 0.05) revealed that edges between arousal and desire, arousal and lubrication, and orgasm and distress were most strongly connected. Overall, edges within symptoms of the same construct were stronger than those between constructs. That is, edges within depression symptoms were stronger than those between depression and sexual function symptoms. One exception was the connection between pain and anhedonia, which was significantly stronger than the majority of connections in the network. Finally, distress was connected with both sexual function and depressive symptoms.

## Anxiety, sexual function, and sexual distress symptoms

### Centrality of nodes and edges

In order, arousal, tension, nervousness, and distress had the greatest centrality in this network. Arousal, tension, and nervousness had the greatest one–step expected influence; tension, nervousness, and arousal also had the greatest two–step expected influence. This means that sexual arousal, tension, and nervousness are central and not only have direct associations with other symptoms, but also have indirect associations with many other symptoms. This can be seen when examining arousal, which is directly connected to all sexual function domains, but also directly linked to fearfulness and feeling scared via distress and pain. The centrality stability coefficient indicated good stability (0.85).

A bootstrapped edge–weight difference test (*α* = 0.05) was used to test edge strengths. The edges between distress and orgasm, and nervousness and tension were strongest, and statistically significantly stronger than other edges in the network. Arousal and desire were also strongly connected. Overall, edges within symptoms of the same construct were stronger than those between constructs. One exception to this, sexual distress and pain were connected with anxiety symptoms, suggesting one pathway for comorbidity between problems with sexual pain and anxiety to be upheld.

## Somatization, sexual function, and sexual distress symptoms

### Centrality, nodes, and edges

Arousal, weakness, distress, orgasm, and pain had the greatest centrality meaning that these nodes were strongly connected to other symptoms. Arousal, weakness, and chest pain had both the greatest one–step and two–step expected influence, indicating that these symptoms not only have direct associations with other symptoms, but also have indirect associations with other variables in the network. The centrality stability coefficient indicated good stability (0.82).

Edges between symptoms of the same construct (e.g., sexual function) were stronger than between constructs (e.g., sexual function and somatization). The strongest edge was between distress and orgasm, and this edge was significantly stronger than any other edge in the network. The next strongest edges were between arousal and desire, and arousal and distress. Once again, distress and pain also show connections with somatization symptoms, suggesting one pathway for comorbidity to be upheld between sexual function problems and somatic disorders.

## Discussion

The present research modelled networks of psychological symptoms, sexual function, and sexual distress to better understand how these symptoms may function together. This is an initial step into the examination of potential mechanisms explaining comorbidity between depressive, anxiety, and somatization symptoms and distressing sexual function problems. Specifically, we addressed critical gaps from previous research and assessed associations at the symptom, rather than the disorder level, allowing us to identify which symptoms may be most important for maintaining the network structure, as well as those that bridge between the constructs. Across our three networks, sexual distress and pain during sex consistently bridged associations between psychological symptoms and other domains of sexual function.

In all networks, sexual distress was a central symptom (i.e., strongly connected to other symptoms), and was one of the few symptoms that bridged (i.e., had edges) psychological symptoms and sexual function domains. That is, while sexual function domains were not often directly or strongly connected to psychological symptoms, they were connected to these symptoms indirectly through sexual distress. This suggests that sexual distress might be a potential path for comorbidity to emerge between psychological symptoms and sexual function domains. One explanation for such a pattern may be that psychological symptoms like rumination may spill over into distress about one's sexuality, which in turn maintains and exacerbates problems with sexual function. Accordingly, sexual distress has been found to mediate associations between women's sexual motivation and self–esteem and their greater pain, depression, and anxiety symptoms (Glowacka et al., 2019; Muise et al., 2018). It follows that targeting sexual distress in treatment for individuals with comorbid sexual dysfunction and psychological disorders may be one way to reduce symptoms throughout the network (i.e., for each disorder). Relatedly, targeting psychological symptoms may benefit sexual function through decreasing sexual distress, consistent with research showing that cognitive behavioural interventions for psychological symptoms incidentally decrease problems with sexual function (Hoyer et al., 2009). Accordingly, cognitive behavioural interventions for sexual dysfunction frequently target sexual distress (e.g., through cognitive restructuring of maladaptive thoughts related to sexual problems or targeting negative affective responses to sex; Mestre-Bach et al., 2022). Moreover, established treatments for sexual dysfunction like mindfulness-based Cognitive Behavioural Therapy are effective both for targeting sexual distress, and depression and anxiety (Brotto et al., 2021;Rashedi et al., 2022; Goldberg et al., 2019; Li et al., 2021). Future research should investigate directionality in the associations between psychological symptoms, sexual function, and sexual distress, which would have important implications for understanding how sexual distress upholds comorbidity and explain the mechanism through which targeting sexual distress may have differential benefits for sexual function and psychological difficulties.

Our depression network showed that sexual arousal, sadness, worthlessness, and distress were central to the network (i.e., had high strength centrality) and directly connected to other symptoms (i.e., had high one–step influence). The centrality of arousal to our network aligns with past research. Indeed, problems with arousal has been proposed as a central mechanism through which sexual function problems lead to distress (Stephenson & Meston, 2015) and depression has been established as a predictor of arousal problems (Soler et al., 2021). Sadness and worthlessness had particularly high centrality related to indirect connections (i.e., two–step expected influence), with worthlessness bridging to sexual function via sexual distress, which aligns with the importance of self-worth and sexual distress to women's sexual function (Glowacka et al., 2019). With regard to edges, we saw that sexual function symptoms remained largely disconnected from depressive symptoms. However, sexual function symptoms were indirectly connected with depressive symptoms through sexual distress. Pain during sex was associated with anhedonia, such that anhedonia was linked to pain above and beyond the effects of other symptoms of depression. It may be these associations capture the loss of pleasure or enjoyment that is characteristic of anhedonia and which may be a cause or consequence of pain during sex.

Arousal, tension, and nervousness had high strength centrality, one–step and two–step expected influence in the anxiety network. Tension and nervousness were directly related to other anxiety symptoms as well as indirectly related to sexual function symptoms through fearfulness, restlessness, and feeling scared, suggesting these symptoms in particular, had far-reaching influence. Regarding edges, pain and sexual distress were most strongly connected with feeling scared and fearful, respectively, suggesting potential paths for symptom contagion across difficulties with sexual function and anxiety. Nevertheless, relative to other connections in the anxiety network (i.e., within constructs) these connections were quite weak. It is surprising, given previous literature finding strong comorbidity between sexual function and anxiety symptoms (e.g., Chisari et al., 2021; Hendrickx et al., 2016) that these constructs were not more closely linked in our network. It may be that our non-clinical sample experienced little variability in anxiety and sexual function symptoms. Indeed, examination of our data supports that over 75 % of our sample reported an anxiety score below the midway point of the scale, so it is possible we were unable to capture potential bridges between the two sets of symptoms typically observed in clinical samples. Another explanation, in line with the Dual Control Model, may be that anxiety may paradoxically increase sexual function in some individuals through transference of physiological arousal to sexual excitation or through the interplay of excitatory and inhibitory pathways (Barlow et al., 1983; Ashbaugh et al., 2022; Janssen & Bancroft, 2023; Kane et al., 2019).

Our somatization network showed that arousal, weakness, distress, and orgasm had high strength centrality and one–step expected influence. Additionally, chest pain had high two–step expected influence. Physiological sexual function symptoms (e.g., arousal and orgasm) were more central than cognitive sexual function symptoms (e.g., desire and satisfaction) in the somatization network, perhaps reflecting the shared physiological nature of these symptoms. With regard to edges, our findings reflect the importance of somatic symptoms to pain during sex relative to other facets of sexual function. Our somatization network showed that pain during sex was connected to poorer sexual function in other domains (especially lubrication) and somatic symptoms like weakness, nausea, and feeling faint. The intuitive connection between lubrication and pain is consistent with research that vaginal dryness is a primary contributor to pain during penetration (Sutton et al., 2012). It is possible that pain during sex was directly associated with symptoms like nausea and weakness, but not cardiovascular symptoms, because this pain implicates central nervous system sensitization and skeletal muscle tension (Kaarbø et al., 2023; Rubal et al., 2023). Additionally, different interoceptive capacities exist for cardiac and gastric sensations, which might explain the importance of some somatic symptoms over others for connecting sexual function and psychological symptoms (Davey et al., 2021).

### What the network approach can tell us about comorbidity

The present study fits into a broader trend to move toward a transdiagnostic approach to psychopathology that accounts for symptoms that cut across traditional diagnostic categories. Research examining relationships between psychopathology and sexual dysfunction has found that transdiagnostic factors like worry and neuroticism have a direct effect on sexual distress, reinforcing support for a cognitive–emotional approach to sexual dysfunction (Tavares et al., 2020; Nobre et al., 2022; Pascoal et al., 2020; Paulus et al., 2016). There is also evidence that depression, anxiety, somatic disorders, and sexual dysfunction might fall under an internalizing dimension that could explain their co–occurrence (Forbes et al., 2016b). This study elucidated relationships between dimensions of sexual function and psychological symptoms (e.g., the role of pain and distress in linking other aspects of sexual function with psychological symptoms), thereby identifying potential bridges between disorders. These findings support that comorbidity between depression, anxiety, somatization, and sexual dysfunction might be related to shared elements between these disorders, above and beyond variability explained by a possible internalizing dimension (Laurent & Simons, 2009). Conversely, theoretical and empirical evidence suggests that these constructs have diverse etiologies and do not necessarily fit under one dimension or latent construct. For example, cardiovascular disease contributes to problems with arousal, which in turn may influence other aspects of sexual response, rather than cardiovascular disease directly causing other sexual function difficulties (Miner et al., 2012). This symptom contagion or connection is best modelled through the network approach such that there is opportunity to see the reciprocal, direct, and indirect links between symptoms, rather than assuming that symptoms are caused by a latent underlying dimension.

### Limitations and future directions

Despite the large sample size and variability in sexual function in our sample and the use of a sophisticated analytic technique, the data were cross–sectional and thus preclude causal claims (Huang et al., 2023). These data came from a primarily Portuguese community sample and individuals were not screened for comorbid sexual dysfunction and psychological disorders. Although some research suggests that Portuguese women may hold relatively positive attitudes toward sex (Træen et al., 2019), there is also evidence that Portuguese women's sexuality is influenced by conservative Catholic traditions and negative sexual beliefs (e.g., a focus on abstinence, sex for procreation and not pleasure; Soares & Nobre, 2012). In the Portuguese context, negative sexual attitudes and beliefs have been associated with poorer sexual function and greater sexual distress (e.g., Nobre et al., 2022; Pascoal et al., 2018), and might be one set of culturally-relevant transdiagnostic factors that influenced our findings.

Additionally, the present research did not consider how other established intrapersonal factors (e.g., sexual victimization, attachment; Bhuptani et al., 2024; Dworkin et al., 2017) and interpersonal factors (e.g., perceived partner responsiveness, dyadic communication; Rosen and Bergeron, 2019) are associated with sexual function problems. Although we did not measure these factors, they might be important drivers connecting psychological symptoms, sexual function, and sexual distress. Together, these limitations prevent us from directly examining alternate explanations for comorbidity between sexual dysfunction and psychological difficulties (e.g., the latent variable model) as well as directionality within the networks. Future longitudinal research, including with clinical samples, is needed to determine whether changes in central symptoms like sexual distress or pain are more likely to be preceded or followed by changes to other sexual function and psychological symptoms. Such approaches might also be informative within treatment studies, where a network analysis might reveal evidence for which symptoms are most effective to target to produce change throughout the network. Additionally, future research might directly compare clinical and non–clinical samples with respect to their network structure to examine similarities and differences between the networks of symptoms.

We found that symptoms tended to cluster within their own constructs (i.e., sexual function symptoms clustered together and psychological symptoms clustered together with few symptoms crossing these boundaries). While such clustering may reflect the true underlying symptom networks, it may have arisen due to shared measurement variance of the FSFI, FSDS–R, and BSI-18 respectively. Future research could use a standardized clinical interview to assess depressive, anxiety, somatization, and sexual function symptoms, mitigating the influence of the measurement structure and variance on the modelled associations within the network.

## Conclusion

The current study provides a novel examination of associations between sexual function, sexual distress, and psychological symptoms using network analysis. Across all networks, sexual distress was an important symptom that bridged sexual function problems and psychological problems. This suggests that sexual distress may be a key factor for the development or maintenance of comorbidities between problems with sexual function and psychological difficulties, and has potential implications for clinical practice, such as emphasizing the assessment and treatment of sexual distress in the presence of comorbid psychological symptomatology. In sum, this research highlights a novel approach to exploring symptom-level interactions between sexual function and psychological variables, as well as the different patterns of association that might exist for individuals with comorbid sexual dysfunction and psychological disorders. Further investigation into the directionality of these relationships, as well as testing in clinical populations, is needed.

## Declaration of competing interest

The authors declare that they have no known competing financial interests or personal relationships that could have appeared to influence the work reported in this paper.
